# Supplementary material for: Specialized heart failure clinics versus primary care: Extended registry-based follow-up of the NorthStar trial
Source: PLoS One. 2023 Jun 8;18(6):e0286307. doi: 10.1371/journal.pone.0286307 (PMC10249840; doi:10.1371/journal.pone.0286307)
Supplement: S1 Methods — (PDF) [file pone.0286307.s001.pdf]

S1 Methods. Inclusion and exclusion criteria.

Eligibility criteria were as follows: age of at least 18 years, a left ventricular ejection fraction (LVEF) of no >45% at the baseline visit in the HFC, educated in HF (=educated in HF and self-management) and on optimal medical therapy with an ACE-I/ARB (unless contraindicated), a BB (unless contraindicated) at the recommended—or the maximally tolerated dose, an ARA (unless contraindicated), and an ICD and/or CRT if indicated. In addition, patients were required to fulfill predefined criteria for clinical stability (=no changes in doses of diuretics, HF symptoms, NYHA class constant, and physical examination without signs of volume overload) at two consecutive visits to the HFC.

Exclusion criteria were: plasma creatinine >200 µmol/L, waiting for a heart transplant, valvular, or ischaemic heart disease with planned surgery or percutaneous intervention, withdrawal of ACE/ARBs, BB, and ARAs due to a reversible cause of cardiomyopathy, malignancy with life expectancy <5 years, and dementia.
