# Supplementary material for: Specialized heart failure clinics versus primary care: Extended registry-based follow-up of the NorthStar trial
Source: PLoS One. 2023 Jun 8;18(6):e0286307. doi: 10.1371/journal.pone.0286307 (PMC10249840; doi:10.1371/journal.pone.0286307)
Supplement: S1 Table — (PDF) [file pone.0286307.s002.pdf]

S1 Table: Three most common primary causes of rehospitalizations (overnight stays only) during follow-up according to the type of disease.

Type of disease	ICD-10 codes	HFC	PC	Total
**Certain infectious and parasitic diseases**	**DA and DB**	**101**	**112**	**223**
Other sepsis	DA41	45	47	92
Other gastroenteritis and colitis of infectious and unspecified origin	DA09	17	15	32
Bacterial infection of unspecified site	DA49	11	13	24
**Neoplasms and hematological diseases**	**DC and DD**	**211**	**210**	**421**
Other anemias	DD64	42	33	75
Malignant neoplasm of bronchus and lung	DC34	13	18	31
Benign neoplasm of colon, rectum, anus, and anal canal	DD12	15	6	21
**Endocrinological diseases**	**DE**	**102**	**112**	**214**
Volume depletion	DE86	34	49	83
Type 2 diabetes mellitus	DE11	23	20	43
Other disorders of fluid, electrolyte, and acid-base balance	DE87	20	18	38
**Cardiovascular diseases**	**DI**	**1164**	**1013**	**2177**
Heart failure	DI50	429	404	833
Atrial fibrillation/flutter	DI48	129	115	244
Chronic ischemic heart disease	DI25	106	100	206
**Respiratory diseases**	**DJ**	**262**	**401**	**663**
Pneumonia, organism unspecified	DJ18	102	152	254
Other chronic obstructive pulmonary disease	DJ44	46	100	146

Respiratory failure, not elsewhere classified	DJ96	37	46	83
**Gastrointestinal diseases**	**DK**	**193**	**205**	**398**
Cholelithiasis	DK80	14	29	43
Inguinal hernia	DK40	15	21	36
Other functional intestinal disorder	DK59	12	21	33
**Skin and subcutaneous diseases**	**DM**	**84**	**105**	**189**
Gonarthrosis	DM17	24	9	33
Other spondylopathies	DM48	<5	17	19
Coxarthrosis	DM16	6	11	17
**Genitourinary diseases**	**DN**	**167**	**125**	**292**
Chronic kidney disease	DN18	43	21	64
Other disorders of the urinary system	DN39	32	25	57
Cystitis	DN30	24	16	40
**Other**		**953**	**909**	**1862**
Medical observation and evaluation for suspected diseases and conditions	DZ03	263	252	515
Care involving use of rehabilitation procedures	DZ50	44	58	102
Syncope and collapse	DR55	56	34	90
