# Supplementary material for: Specialized heart failure clinics versus primary care: Extended registry-based follow-up of the NorthStar trial
Source: PLoS One. 2023 Jun 8;18(6):e0286307. doi: 10.1371/journal.pone.0286307 (PMC10249840; doi:10.1371/journal.pone.0286307)
Supplement: S2 Table — (PDF) [file pone.0286307.s003.pdf]

S2 Table. The 8-year hazard rate of all-cause death, cardiovascular death, cardiovascular death or heart failure, and heart failure among 2-year survivors in groups defined by the number of prior hospitalizations for heart failure within the first two years following randomization.

Variable	Hazard ratio [95% CI], p-value			
	All-cause death	Cardiovascular death	Cardiovascular death or heart failure	Heart failure
PC vs HFC	1.01 [0.83-1.24], p = 0.90	1.00 [0.79-1.28], p = 0.97	0.97 [0.82-1.15], p = 0.73	1.01 [0.84-1.22], p = 0.88
HF				
0	Ref.	Ref.	Ref.	Ref.
1	1.95 [1.48-2.56], p <0.001	2.14 [1.56-2.93], p <0.001	1.79 [1.42-2.26], p <0.001	1.80 [1.38-2.34], p <0.001
>1	2.85 [2.17-3.73], p <0.001	2.86 [2.08-3.94], p <0.001	2.63 [2.06-3.35], p <0.001	2.94 [2.25-3.84], p <0.001
Interaction (PC vs HFC)				
HF = 0	1.00 [0.78-1.29], p = 0.98	0.99 [0.73-1.34], p = 0.93	1.06 [0.86-1.30], p = 0.59	1.08 [0.86-1.22], p = 0.49
HF = 1	0.74 [0.45-1.23], p = 0.25	0.79 [0.45-1.40], p = 0.42	0.76 [0.49-1.17], p = 0.22	0.83 [0.51-1.34], p = 0.45
HF >1	1.40 [0.87-2.26], p = 0.16	1.36 [0.78-2.39], p = 0.28	0.84 [0.54-1.31], p = 0.45	0.92 [0.57-1.49], p = 0.75

Abbreviations:

95% CI = 95% confidence intervals, PC = primary care, HFC = heart failure clinic, HF = number of hospitalizations for heart failure within two years following randomization.
