# Supplementary figures and images for: Specialized heart failure clinics versus primary care: Extended registry-based follow-up of the NorthStar trial
Source: PLoS One. 2023 Jun 8;18(6):e0286307. doi: 10.1371/journal.pone.0286307 (PMC10249840; doi:10.1371/journal.pone.0286307)

S1 Figure. Flowchart.

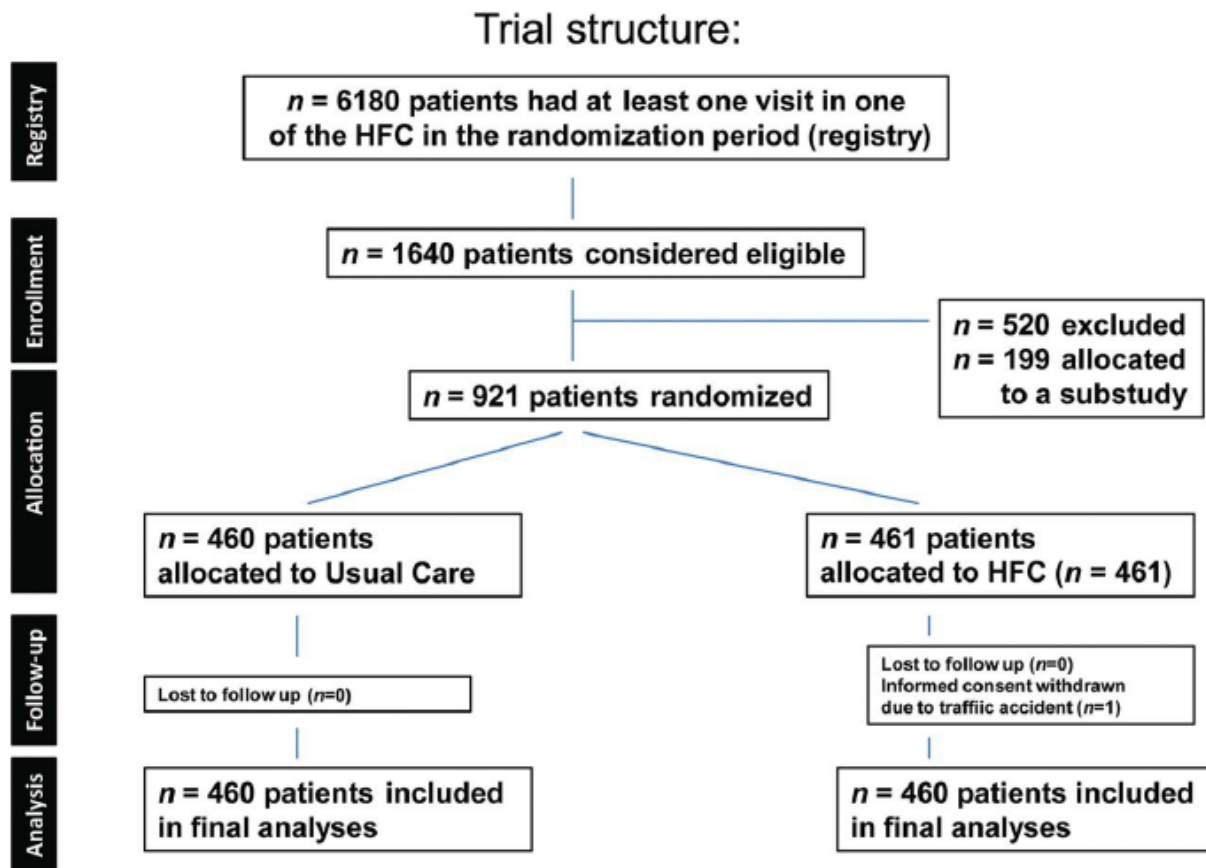

Abbreviations:

HFC = Heart Failure clinic.

Supplement: S1 Fig — (PDF) [file pone.0286307.s004.pdf]
