# Supplementary material for: Specialized heart failure clinics versus primary care: Extended registry-based follow-up of the NorthStar trial
Source: PLoS One. 2023 Jun 8;18(6):e0286307. doi: 10.1371/journal.pone.0286307 (PMC10249840; doi:10.1371/journal.pone.0286307)
Supplement: S2 Fig — (PDF) [file pone.0286307.s005.pdf]

S2 Figure. The 8-year absolute risk of all-cause death, cardiovascular death, discharge for heart failure, and cardiovascular death or discharge for heart failure in 2-year survivors in groups defined by the number of prior discharge diagnoses for heart failure.

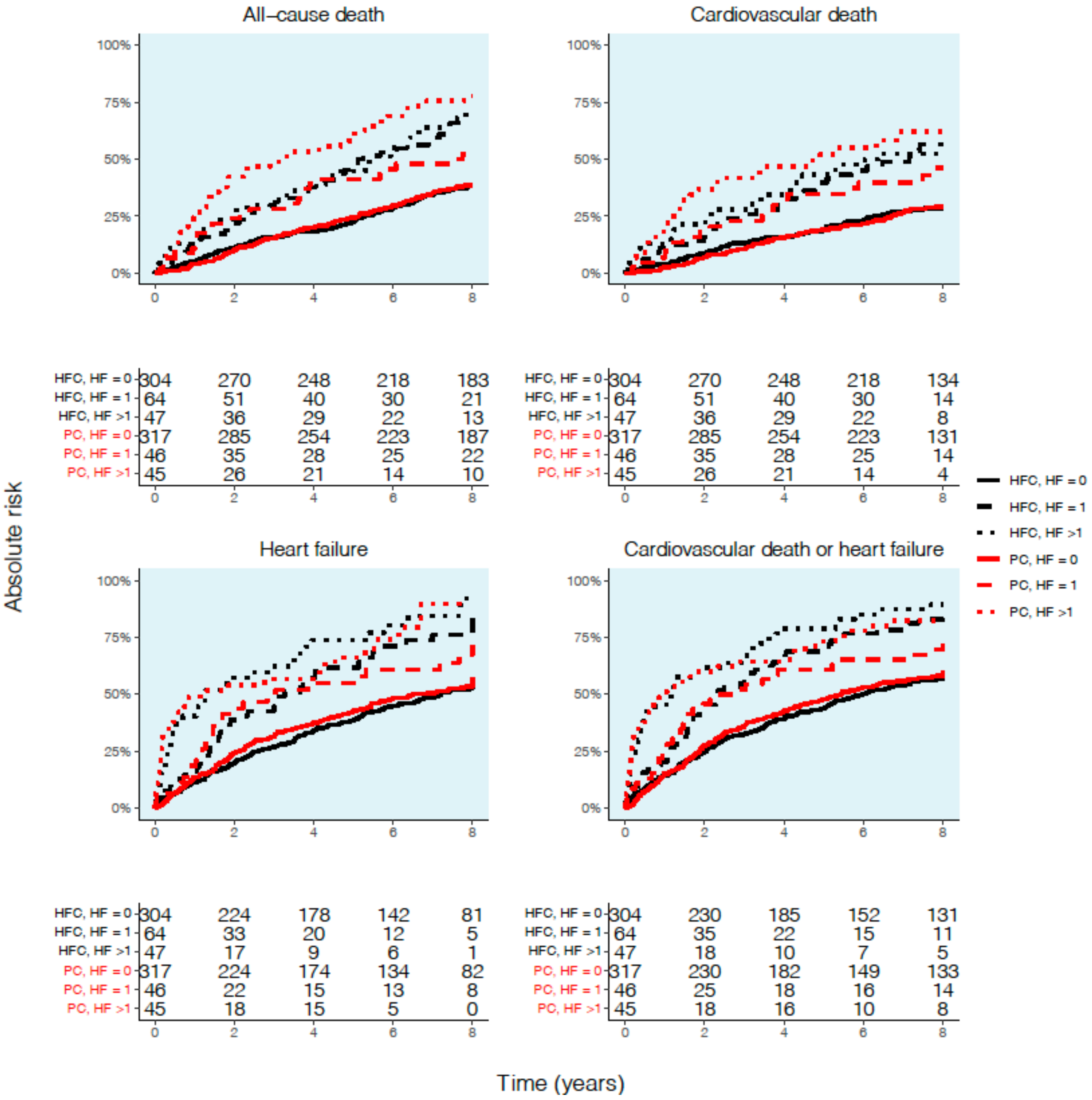

<u>Abbreviations:</u>
HFC = Heart failure clinic, PC = primary care, HF = the number of prior discharge diagnoses for heart failure within the first two years from randomization.
