# Supplementary material for: Specialized heart failure clinics versus primary care: Extended registry-based follow-up of the NorthStar trial
Source: PLoS One. 2023 Jun 8;18(6):e0286307. doi: 10.1371/journal.pone.0286307 (PMC10249840; doi:10.1371/journal.pone.0286307)
Supplement: Attachment — Submitted filename: Response ot reviewers.docx [file pone.0286307.s006.docx]

**Editors/Reviewers' Comments:**

In the following reply, the reviewers’ comments are formatted in bold, black color (**reviewers’ comments**). The comments are followed by our reply in normal, blue color (our reply). When changes to the manuscript are made, we have printed the previous version in normal, black color (previous version) with all changes clearly marked by use of normal, green color for insertions (inserted text), whereas text removed from the manuscript is in normal, strikethrough, red color (~~removed text~~).

# **Additional Editor Comments:** **Comment 1.1. This is an interesting study. It would be interesting to account for additional events during follow-up. Please include an additional analysis comparing groups based on number of HF re-hospitalizations. Response to comment 1.1:** Thank you for your important comments. We have included a new analysis comparing groups based on number of HF-rehospitalizations as requested. We chose to examine 2-year survivors, since all 2-year survivors remained in the randomization at 2 years following randomization. In 2-year survivors, patients were followed from 2 years following randomization until the event of interest, death or 10 years following randomization. The absolute 8-year risk of the primary and secondary endpoints was estimated in groups defined by the number of HF hospitalizations (0, 1 or >1 HF hospitalizations) within the first 2 years following randomization using the Aalen-Johansen estimator, incorporating the competing risks of death (Kaplan-Meier for all-cause death). We used a Cox regression model adjusted for numbers of HF hospitalizations to calculate hazard ratios with 95% confidence intervals of the primary and secondary outcomes comparing the PC and HFC group. To analyze the main exposure in groups by the number prior HF hospitalization, the interaction between the main exposure (PC vs HFC) and the number prior HF hospitalizations was analyzed in the same Cox models. At 2 years following randomization, 415 (HF = 0, 304 patients; HF = 1, 64 patients; HF >1, 47 patients) and 408 patients (HF = 0, 317 patients; HF = 1, 46 patients; HF >1, 45 patients) were alive in the HFC and PC group, respectively. We observed that the number of prior HF hospitalizations were strongly associated with the primary and secondary outcomes in both the PC and HFC group (please the figure and table below). In the interaction analyses, no differences were observed between the PC and HFC group in subgroups of number of HF hospitalization regardless of the outcome of interest (please see table below). However, caution should be made in the interpretation of these results, since the numbers of both patients and outcomes were quite low, particularly in the groups of patients with prior HF hospitalizations within the first 2 years following randomization. The following figure and table have been included as supplemental analyses.

# S2 Figure. 8-year absolute risk of all-cause death, cardiovascular death, discharge for heart failure, and cardiovascular death or discharge for heart failure in 2-year survivors in groups defined by the number of prior discharge diagnoses for heart failure.

Abbreviations:
HFC = Heart failure clinic, PC = primary care, HF = the number of prior discharge diagnoses for heart failure within the first two years from randomization.

# S1 Table. 8-year hazard rate of all-cause death, cardiovascular death, cardiovascular death or heart failure, and heart failure among 2-year survivors in groups defined by the number of prior hospitalizations for heart failure within the first two years following randomization.

| Variable | Hazard ratio [95% CI], p value | | | |
| --- | --- | --- | --- | --- |
|  | All-cause death | Cardiovascular death | Cardiovascular death or heart failure | Heart failure |
| PC vs HFC | 1.01 [0.83-1.24],  p = 0.90 | 1.00 [0.79-1.28],  p = 0.97 | 0.97 [0.82-1.15],  p = 0.73 | 1.01 [0.84-1.22],   p = 0.88 |
| HF  0  1  >1 | Ref.  1.95 [1.48-2.56],  p <0.001  2.85 [2.17-3.73],  p <0.001 | Ref.  2.14 [1.56-2.93],  p <0.001  2.86 [2.08-3.94],  p <0.001 | Ref.  1.79 [1.42-2.26],  p <0.001  2.63 [2.06-3.35],  p <0.001 | Ref.  1.80 [1.38-2.34],  p <0.001  2.94 [2.25-3.84],  p <0.001 |
|  | | | | |
| Interaction  (PC vs HFC) |  |  |  |  |
| HF = 0 | 1.00 [0.78-1.29],  p = 0.98 | 0.99 [0.73-1.34],  p = 0.93 | 1.06 [0.86-1.30],  p = 0.59 | 1.08 [0.86-1.22],  p = 0.49 |
| HF = 1 | 0.74 [0.45-1.23],  p = 0.25 | 0.79 [0.45-1.40],  p = 0.42 | 0.76 [0.49-1.17],  p = 0.22 | 0.83 [0.51-1.34],  p = 0.45 |
| HF >1 | 1.40 [0.87-2.26],  p = 0.16 | 1.36 [0.78-2.39],  p = 0.28 | 0.84 [0.54-1.31],  p = 0.45 | 0.92 [0.57-1.49],  p = 0.75 |

Abbreviations:
95% CI = 95% confidence intervals, PC = primary care, HFC = heart failure clinic, HF = number of hospitalizations for heart failure within two years following randomization.

**Comment 1.2. Please check figure 1, the titles on the KM curves seem not corresponding to the figure caption.
Response to comment 1.2:** Thank you, this has been corrected.

 **Comment 1.3. Please better comment on the borderline P for interaction for AF in Fig 2. Statistical significance could be missing simply due to power issues.
Response to comment 1.3:** This has been corrected.  **Comment 1.4. Can the authors provide more information on the reasons for re-hospitalizations during the follow-up?
Response to comment 1.4:** Thank you for this important comment. We have provided a subgroup analysis in the present study for the primary outcome, cardiovascular death or discharge for heart failure, as shown in Figure 2. However, we are unsure whether more variables are requested to the existing figure, or whether a new analysis is being requested (e.g., baseline characteristics in 2 year survivors according to the main exposure and the number of prior HF hospitalizations). We will happily provide with more information based on what is being requested.
 **Comment 1.5. The rates of device therapy at randomization is very low. It would be important to have info during follow-up.
Response to comment 1.5:** We have provided with information on devices in 5-year survivors.

**Changes in the manuscript:**

From Results:
The baseline characteristics among the 921 patients included have been described previously (*Table 1*).(2) After 5 years of follow-up, 335 (72.7%) patients in the HFC group and 325 (70.7%) patients in the PC group were still alive. At 5 years following randomization, 64 (19.1%) and 62 (19.1%) patients had an implantable cardioverter-defibrillator in the HFC and PC group, respectively (chisq test p value = 1.00), and 25 (7.5%) and 30 (9.2%) patients had a cardiac resynchronization therapy device in the HFC and PC group, respectively (chisq test p value = 0.50).
 **Comment 1.6. Please add more details on the initial trial. The paper must be self-standing and the readers/reviewers should not go searching for other info from different prior publications.**

**Response to comment 1.6:** Thank you, more information on the initial trial has been added.

[Note: HTML markup is below. Please do not edit.]

Reviewers' comments:

Reviewer's Responses to Questions

**Comments to the Author**

1. Is the manuscript technically sound, and do the data support the conclusions?

The manuscript must describe a technically sound piece of scientific research with data that supports the conclusions. Experiments must have been conducted rigorously, with appropriate controls, replication, and sample sizes. The conclusions must be drawn appropriately based on the data presented.

Reviewer #1: Yes

Reviewer #2: Yes

Reviewer #3: Yes

2. Has the statistical analysis been performed appropriately and rigorously?

Reviewer #1: Yes

Reviewer #2: Yes

Reviewer #3: Yes

3. Have the authors made all data underlying the findings in their manuscript fully available?

The [PLOS Data policy](http://www.plosone.org/static/policies.action#sharing) requires authors to make all data underlying the findings described in their manuscript fully available without restriction, with rare exception (please refer to the Data Availability Statement in the manuscript PDF file). The data should be provided as part of the manuscript or its supporting information, or deposited to a public repository. For example, in addition to summary statistics, the data points behind means, medians and variance measures should be available. If there are restrictions on publicly sharing data—e.g. participant privacy or use of data from a third party—those must be specified.

Reviewer #1: No

Reviewer #2: No

Reviewer #3: No

**Response to comment:**

The following text has been added to the data availability statement:

Due to restrictions related to Danish law and protecting patient privacy, the combined set of data utilized in this present study can only be made available through a trusted third party, Statistics Denmark. Requests for data may be sent to Statistics Denmark at: <http://www.dst.dk/en/OmDS/organisation/TelefonbogOrg.aspx?kontor=13&tlfbogsort=sektion> or the Danish Data Protection Agency: <https://www.datatilsynet.dk/english/contact-us>

4. Is the manuscript presented in an intelligible fashion and written in standard English?

PLOS ONE does not copyedit accepted manuscripts, so the language in submitted articles must be clear, correct, and unambiguous. Any typographical or grammatical errors should be corrected at revision, so please note any specific errors here.

Reviewer #1: Yes

Reviewer #2: Yes

Reviewer #3: Yes

5. Review Comments to the Author

Please use the space provided to explain your answers to the questions above. You may also include additional comments for the author, including concerns about dual publication, research ethics, or publication ethics. (Please upload your review as an attachment if it exceeds 20,000 characters)

**Reviewer #1: PONE-D-22-18899: statistical review**

**Comment 2.1. This study compares long-term survival between heart failure patients who have been randomly assigned to follow-up in a specialized HF clinic or primary care. 5-year adherence to prescribed neurohormonal blockade in 5-year survivors is also assessed. Traditional survival analysis methods are correctly exploited to compare the survival trajectories between the two groups and beta regression is correctly considered for comparing adherence proportions. I have nothing to say about this paper: the research question is well posed, statistical methods are appropriate, results are correctly interpreted and possible limitations of the study are nicely remarked in the discussion.**

**Response to comment 2.1:** Thank you for your comments.

**Reviewer #2: 
In this manuscript, authors report findings from the extended follow-up of a trial conducted in Denmark (NorthStar) that randomized patients with heart failure with reduced ejection fraction (HFrEF) to be followed up in primary care or in a specialized HF clinic once treatment had been optimized.

FU data were obtained using linked electronic health records.

Overall, the manuscript is clear and easy to read till the end.

However, I think that the main limitation of this study, as acknowledged by the authors, is that during a period of up to 10 years many bad things can happen to patients with heart failure, and a substantial proportion of those randomized to a primary care clinic follow-up would have received specialist input due to clinical deterioration which, in my opinion, limits the relevance of these findings.

Comment 3.1. Authors might add a comment to adherence to beta-blockers, which seems higher that that to RASi.**

**Response to comment 3.1:** Thank you for your comments. We have added a comment on the adherence to BB and RASi in the discussion section.

**Changes in the manuscript:**

From Adherence to guideline therapy in the Discussion section:

In patients who were alive after 5 years, we evaluated the 5-year adherence of HF therapy. Here we did not observe any difference between groups. Considering that specialized HF clinics are designed to improve adherence, this finding was surprising. However, it supports the overall neutral result considering the lack of effect of the patient education *per se* observed in the COACH trial.(15) It may be further explained by the fact that the patients were already on optimal medical therapy and all patients were educated in self-care and disease management. It should be noted that the adherence to MRAs was poor in both groups. MRAs improve life expectancy considerably(16,17) and more focus on adherence to MRA is needed in future studies together with implementation of sacubitril-valsartan(18) and sodium glucose inhibitors.(19,20) Somewhat surprisingly, the proportion of patients with a PDC-level >80% appeared to be slightly higher BB compared to RASi. However, it should be mentioned that the patients in the present study were made sure to tolerate the HF medication to the max tolerable dose before randomization, which may have ensured relatively high adherence to BB. Additionally, medications that are prescribed more than once daily complicates the calculation of PDC. Thus, calculations of PDC and differences in adherence between drugs should be interpreted with caution.

**Reviewer #3: 
The authors present a very interesting study on the value of specialized heart failure clinics versus primary care. It is a relevant study, but I have some comments:
Comment 4.1. Although an NT-proBNP > 1000 pg/mL might identify high-risk patients, an HF hospitalization (43% in the initial study) the previous year might be a marker of higher risk than NTproBNP values.
Response to comment 4.1:** Thank you for your comments. We have included a new analysis comparing groups based on number of HF-rehospitalizations as requested by editor. Please see response to comment 1.1.

**Comment 4.2. The authors use both MRA and ARA. ARA is not defined in the Abbreviation nor Table 1. Interestingly, the use of MRA appears to increase from randomization (33%) to the 5-year follow-up MRA (HFC: 51.9%; PC: 52.0%). It may be associated with the publication of pivotal MRA trials in HF. However, it is surprising that PC prescribed MRA at a relatively high percentage.
Response to comment 4.2:** We agree with your comments. The increase in the use of MRAs and ARAs is likely due to the increased use with calendar time. This increase is expected, however it is rather surprising that PC prescribed MRAs at a similar percentage to the HFC group, and that the adherence to MRA was similar in both groups. Table 1 has been corrected. **Comment 4.3. Was CRT or ICD different at 5-year follow-up? The use at baseline is surprisingly low.
Response to comment 4.3:** We have provided with information on devices in 5-year survivors as shown in response to comment 1.5.

6. PLOS authors have the option to publish the peer review history of their article ([what does this mean?](https://journals.plos.org/plosone/s/editorial-and-peer-review-process#loc-peer-review-history)). If published, this will include your full peer review and any attached files.

If you choose “no”, your identity will remain anonymous but your review may still be made public.

**Do you want your identity to be public for this peer review?** For information about this choice, including consent withdrawal, please see our [Privacy Policy](https://www.plos.org/privacy-policy).

Reviewer #1: No

Reviewer #2: No

Reviewer #3: No
