# Supplementary material for: Specialized heart failure clinics versus primary care: Extended registry-based follow-up of the NorthStar trial
Source: PLoS One. 2023 Jun 8;18(6):e0286307. doi: 10.1371/journal.pone.0286307 (PMC10249840; doi:10.1371/journal.pone.0286307)
Supplement: Attachment — Submitted filename: Response ot reviewers2.docx [file pone.0286307.s007.docx]

**Editors/Reviewers' Comments:**

In the following reply, the reviewers’ comments are formatted in bold, black color (**reviewers’ comments**). The comments are followed by our reply in normal, blue color (our reply). When changes to the manuscript are made, we have printed the previous version in normal, black color (previous version) with all changes marked by the use of normal, green color for insertions (inserted text), whereas text removed from the manuscript is in normal, strikethrough, red color (~~removed text~~).

# **Additional Editor Comments: The authors provided a properly revised version of the manuscript with almost all points addressed. Comment 1.1. There is still 1 of my previous points to be addressed, specifically point 1.4 in which I did not ask for specific new analyses but simply a table or text reporting reasons for re-hospitalizations during follow-up (new acute coronary syndrome, infections, diuretic resistance, worsening of renal function etc.). Response to comment 1.1:** Thank you for your comments. We have included a table with the 3 most common causes of rehospitalizations during follow-up for a range of diseases according to the table shown below. Only primary causes of an overnight stay were considered.

**Table S1: Three most common primary causes of rehospitalizations (overnight stays only) during follow-up according to the type of disease.**

| **Type of disease** | **ICD-10 codes** | **HFC** | **PC** | **Total** |
| --- | --- | --- | --- | --- |
| **Certain infectious and parasitic diseases** | **DA and DB** | **101** | **112** | **223** |
| Other sepsis | DA41 | 45 | 47 | 92 |
| Other gastroenteritis and colitis of infectious and unspecified origin | DA09 | 17 | 15 | 32 |
| Bacterial infection of unspecified site | DA49 | 11 | 13 | 24 |
| **Neoplasms and hematological diseases** | **DC and DD** | **211** | **210** | **421** |
| Other anemias | DD64 | 42 | 33 | 75 |
| Malignant neoplasm of bronchus and lung | DC34 | 13 | 18 | 31 |
| Benign neoplasm of colon, rectum, anus, and anal canal | DD12 | 15 | 6 | 21 |
| **Endocrinological diseases** | **DE** | **102** | **112** | **214** |
| Volume depletion | DE86 | 34 | 49 | 83 |
| Type 2 diabetes mellitus | DE11 | 23 | 20 | 43 |
| Other disorders of fluid, electrolyte, and acid-base balance | DE87 | 20 | 18 | 38 |
| **Cardiovascular diseases** | **DI** | **1164** | **1013** | **2177** |
| Heart failure | DI50 | 429 | 404 | 833 |
| Atrial fibrillation/flutter | DI48 | 129 | 115 | 244 |
| Chronic ischemic heart disease | DI25 | 106 | 100 | 206 |
| **Respiratory diseases** | **DJ** | **262** | **401** | **663** |
| Pneumonia, organism unspecified | DJ18 | 102 | 152 | 254 |
| Other chronic obstructive pulmonary disease | DJ44 | 46 | 100 | 146 |
| Respiratory failure, not elsewhere classified | DJ96 | 37 | 46 | 83 |
| **Gastrointestinal diseases** | **DK** | **193** | **205** | **398** |
| Cholelithiasis | DK80 | 14 | 29 | 43 |
| Inguinal hernia | DK40 | 15 | 21 | 36 |
| Other functional intestinal disorder | DK59 | 12 | 21 | 33 |
| **Skin and subcutaneous diseases** | **DM** | **84** | **105** | **189** |
| Gonarthrosis | DM17 | 24 | 9 | 33 |
| Other spondylopathies | DM48 | <5 | 17 | 19 |
| Coxarthrosis | DM16 | 6 | 11 | 17 |
| **Genitourinary diseases** | **DN** | **167** | **125** | **292** |
| Chronic kidney disease | DN18 | 43 | 21 | 64 |
| Other disorders of the urinary system | DN39 | 32 | 25 | 57 |
| Cystitis | DN30 | 24 | 16 | 40 |
| **Other** |  | **953** | **909** | **1862** |
| Medical observation and evaluation for suspected diseases and conditions | DZ03 | 263 | 252 | 515 |
| Care involving use of rehabilitation procedures | DZ50 | 44 | 58 | 102 |
| Syncope and collapse | DR55 | 56 | 34 | 90 |

# **Comment 1.2. Also, few points from reviewer 4 should be addressed. Response to comment 1.2:** Comments from reviewer 4 have been addressed below.

**Reviewer #4: In this manuscript, Dr. Malmborg and colleagues tried to assess if there is a difference in hospitalization for heart failure or cardiovascular mortality comparing specialized heart failure clinics versus primary care through an extended post-hoc registry-based follow-up study of a randomized controlled trial.
Overall, this is a quite nicely written article, greatly improved already by previous revisions, with a clinical relevance.
However, some relevant issues need to be addressed by the authors:**

**Comment 2.1: Albeit the study was conducted rigorously, the result is in contrast to recent results from registry studies involving far larger numbers of patients (https://onlinelibrary.wiley.com/doi/full/10.1002/ehf2.13848 ; https://www.internationaljournalofcardiology.com/article/S0167-5273(21)01334-6/fulltext). This should be expanded upon in the discussion section, pointing out why there may be this discrepancy.**

**Response to comment 2.1: Thank you for your important comments. Both studies were based on observational data from HF patients included in the Swedish HF Registry between 2000-2016.(1,2) In contrast to our study, both studies observed a lower mortality rate and a higher rate of HF hospitalizations in the HFC compared to the PC group. Although their data is large and their results are comprehensively adjusted from a well-characterized population, their results may have been affected by residual confounding and imputed data, in which missing data may have influenced their results. Secondly, the Swedish HF Registry uses rather broad inclusion criteria, and it is not fully clear whether all patients were included following fully optimized medical treatment in an HFC, both factors limiting the comparison to our study. Lastly, Lindberg et al.(2) included HFmrEF and HFpEF patients further limiting the comparison to our study. We have included these comments in our discussion.**

***From “Other studies and subgroups” in the Discussion section:***

In contrast to our study, two recent studies using observational data from the Swedish HF Registry observed a lower mortality rate and a higher rate of HF hospitalizations in the HFC compared to the PC group.(13,14) However, their results may have been affected by residual confounding and imputed data. Secondly, the Swedish HF Registry uses a broad inclusion criterion, and it is not fully clear whether all patients were included following fully optimized medical treatment in an HFC, both factors limiting the comparison to our study. According to our knowledge, no previous trials have evaluated 10 years of follow-up in two different parts of a public health care system in a randomized clinical trial setting. We did not observe any additional effect of follow-up in a specialized HF clinic. This may be explained by several factors. First, there is a possibility of selection bias at enrolment. Patients included in a randomized clinical trial are generally healthier than patients in general,(13) and it cannot be ruled out that a sicker population would have benefitted from continued follow-up. Second, the intervention was too weak considering that the patients were already receiving optimal medical therapy before randomization leaving only little room for further optimization. Third, cross over between the two arms. Within the original study period, only minor cross-over occurred,(2) but during a 10 years period with a high proportion of HF re-hospitalizations, it may have occurred over time. Finally, no statistical interactions between treatment allocation and traditional subgroups on the risk of the primary outcome were observed, supporting the overall neutral result. Therefore, more sophisticated risk stratification than the identification of subgroups is probably needed in the future to identify patients that need specialized care for years.(14)

**Comment 2.2: Table 1: The authors report an haemoglobin mean level of 8.6, but only about 20% of patients with anemia. How is this possible? What are the upper letter a in anemia and upper letter b in hyponatremia for?**

**Response to comment 2.2: Unfortunately, the unit for hemoglobin was written as g/dL, but was supposed to be written as mmol/L. This has been corrected. We have added an explanation of the upper letter a and b below Table 1.**

**Comment 2.3: Figure 2: On the bottom near the hazard ratio should be specified whether left or right is in favour of HFC or PC**

**Response to comment 2.3: This has been added to the figure.**

**Comment 2.4: Please note that the text requires a careful proofreading, since there are several grammar lapses**

**Response to comment 2.4: Thank you. The manuscript has gone through careful proofreading.**

**References**

1.	Kapelios CJ, Canepa M, Benson L, Hage C, Thorvaldsen T, Dahlström U, et al. Non-cardiology vs. cardiology care of patients with heart failure and reduced ejection fraction is associated with lower use of guideline-based care and higher mortality: Observations from The Swedish Heart Failure Registry. Int J Cardiol. 2021 Nov 15;343:63–72.

2.	Lindberg F, Lund LH, Benson L, Schrage B, Edner M, Dahlström U, et al. Patient profile and outcomes associated with follow-up in specialty vs. primary care in heart failure. ESC Heart Fail. 2022 Apr;9(2):822–33.
